# ﻿The brown copper moth, *Tridentaforma browncopper*: DNA barcoding reveals a second species in the family Tridentaformidae (Lepidoptera, Adeloidea)

**DOI:** 10.3897/zookeys.1257.158827

**Published:** 2025-10-24

**Authors:** Spencer K. Monckton, Valerie Levesque-Beaudin, Ken A. Thompson, Aidan Barnes, Kami French, Hunter Hampton, Paul D. N. Hebert

**Affiliations:** 1 Centre for Biodiversity Genomics, University of Guelph, 50 Stone Road East, Guelph, Ontario, N1G 2W1, Canada University of Guelph Guelph Canada; 2 Teck Resources Limited, Suite 3300, Bentall 5, 550 Burrard Street, Vancouver, B.C., V6C 0B3, Canada Teck Resources Limited Vancouver Canada; 3 K’en T’em Limited Partnership, 2954B Shackelly Road, Merritt, B.C., V1K 1B8, Canada K’en T’em Limited Partnership Merritt Canada

**Keywords:** Biotic survey, micromoths, mining industry, Nlaka’pamux traditional territory, Thompson Plateau

## Abstract

A biodiversity monitoring program in south-central British Columbia, Canada, at sites on traditional nłeʔképmx territory near the Highland Valley Copper mine, collected many specimens of an unknown moth. DNA barcode analysis revealed its affinity but deep divergence (14.3%) from *Tridentaforma fuscoleuca* (Braun, 1923), the only species in the family Tridentaformidae. The new species was assigned to the BIN (Barcode Index Number) BOLD:AFK8960. Morphological study confirmed its placement in this family but revealed marked genitalic differences from *T. fuscoleuca*. Given its genetic and morphological divergence, we describe *Tridentaforma browncopper* Monckton & Levesque-Beaudin, **sp. nov.** The scientific name is a translation of the name, skʷúnkʷl̓itkax̣n̓I, chosen by nłeʔképmx Elders and Knowledge Keepers, which means “brown copper moth”. Its discovery and naming reflect an effective collaboration among biodiversity scientists, industry representatives, and Indigenous communities. It also demonstrates how DNA barcoding can facilitate species descriptions without requiring taxonomists with specialist expertise in the group under investigation.

## ﻿Introduction

Nearly 100,000 arthropod specimens were collected during a biodiversity monitoring program on traditional nłeʔképmx territory in south-central British Columbia, near the Highland Valley Copper mine site administered by Teck Resources Limited (Teck). These specimens were sent to the Centre for Biodiversity Genomics (CBG) for barcode analysis (Hebert et al. 2003), work that revealed 3,196 species including over 343 new to the Barcode of Life Data Systems (BOLD) database (Ratnasingham and Hebert 2007). These new taxa included one highly divergent lepidopteran lineage whose DNA barcode sequences suggested its membership in Tridentaformidae Davis, 2015, a family previously known from a single species—*Tridentaforma fuscoleuca* (Braun, 1923) (Regier et al. 2015). This observation provoked deeper investigation, work which indicated that the new BIN (Barcode Index Number; Ratnasingham and Hebert 2013) represented an undescribed species of *Tridentaforma* Davis, 1978.

Details of the discovery were shared with the K’en T’em Limited Partnership, who supported the biodiversity monitoring program, and the Citxw Nlaka’pamux Assembly (CNA), who were asked to name the new species. After consultation with nłeʔképmx Elders and Knowledge Keepers, the species was given the nłeʔképmxcín name skʷúnkʷl̓itkax̣n̓I (approximate romanization: shkwoon-kwleet-kaxh-nee), which translates to English as “brown copper moth” (CNA 2024).

Here, we formally describe the brown copper moth as *Tridentaforma browncopper* Monckton & Levesque-Beaudin, sp. nov.

## ﻿Methods

**Figure 1.**
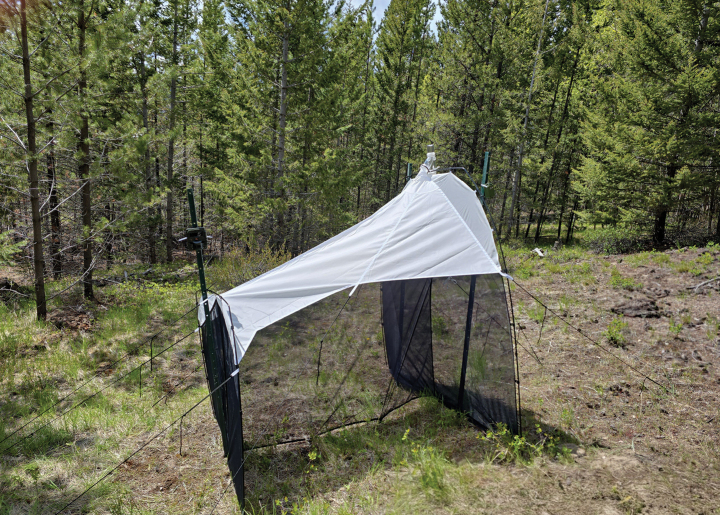
Type locality of *Tridentaforma browncopper* Monckton & Levesque-Beaudin, sp. nov., including deployed Malaise trap. The collection site is a grassy clearing amidst Douglas fir-dominated forest (*Pseudotsuga menziesii* (Mirbel) Franco). Photo courtesy of Reid Harrop.

Seventeen Malaise traps were deployed at six sites near Logan Lake, British Columbia as one component of a biodiversity monitoring program at Highland Valley Copper (HVC), which is owned/operated by Teck. Producing both copper and molybdenum, HVC is the largest open-pit copper mine in Canada. Sampling occurred from July 18–October 31, 2023, and July 29–October 21, 2024. The sites included two recently reclaimed (<5 y) waste-rock sites, two sites adjacent to tailings ponds and reclaimed as agricultural grassland for >20 y, and two unmined reference sites, one of which was subject to a forest fire two years prior. Each site had three traps except the fire site, which had two. Most (93%, *n* = 168) of the 180 *Tridentaforma* specimens were collected at the unburned reference site (Fig. 1), but a few derived from the waste-rock (*n* = 5), tailings (*n* = 6), and fire (*n* = 1) sites. Voucher specimens are stored in 95% EtOH in microwell plates at the CBG except as otherwise indicated.

Bulk Malaise trap samples were sent to the CBG where they were DNA barcoded using standard protocols (deWaard et al. 2019). The DNA extract from each specimen was amplified for the 658 bp barcode region of cytochrome *c* oxidase I (COI; Hebert et al. 2003) and the resultant sequences were uploaded to BOLD (Ratnasingham and Hebert 2007; Ratnasingham et al. 2024), the Barcode of Life Data Systems (https://boldsystems.org/). BOLD subsequently assigned each sequence to a BIN, a species proxy (Ratnasingham and Hebert 2013). In total, the 34,565 specimens that yielded DNA barcodes were assigned to 3,196 different BINs, of which 343 represented first records for BOLD. Among these new BINs, one (BOLD:AFK8960) included 180 records whose nearest identified match was a member of the monotypic family Tridentaformidae (Lepidoptera). To confirm its assignment to this family, genitalic dissections were made on three males and three females. Because DNA extracts were prepared using alkaline lysis (Ivanova et al. 2006), no further tissue clearing was needed. Dissected parts were suspended in glycerin and stored in genitalia vials; genitalia from the holotype, allotype, and imaged paratypes were subsequently slide-mounted in Euparal. In addition, 18 specimens (including those dissected) were critical point dried and mounted on points to examine external morphology. Specimens were imaged and measured using a Keyence (Osaka, Japan) VHX-7000 digital microscope system.

A neighbour-joining tree was generated for all available Tridentaformidae records using the BOLD workbench (Ratnasingham et al. 2024), with distance model set to Kimura 2-parameter and alignment method set to BOLD Aligner. Bootstrap support values were computed for the resulting alignment and topology using phangorn (Schliep 2011) based on 100 replicates.

The holotype and allotype are deposited in the Canadian National Collection of Insects, Arachnids, and Nematodes (**CNC**) in Ottawa while other paratypes are deposited at the CBG (**BIOUG**). All COI sequences are publicly available in BOLD in the dataset DS-BRWNCP (https://doi.org/10.5883/DS-BRWNCP) and are also available in GenBank (https://www.ncbi.nlm.nih.gov/genbank/) via accession numbers PX223413–PX223608 and JN301424.

## ﻿Results

### ﻿ Tridentaforma

Taxon classificationAnimaliaLepidopteraTridentaformidae

Davis, 1978

5F240ADB-0C04-5588-A6A7-A28BA416E99F

Figs 2, 3, 4, 5, 6

Tridentaforma browncopper Monckton & Levesque-Beaudin, sp. nov.

#### Type species.

*Lampronia fuscoleuca* Braun, 1923.

#### Diagnosis.

The genus is distinguished by a trident-like male phallus (Fig. 3A) with slender lateral branches about half as long as the median branch (or nearly so), and by a broad valva bearing three or four discrete, transverse rows of spines (pectens) along its ventral margin (Fig. 3B).

#### Comments.

Traits are mostly consistent with the original generic description (Davis 1978). The following modifications accommodate traits in *T. browncopper*: antenna approximately 0.5–0.8× length of forewing; eye index approximately 1.0–1.2; labial palp with apical segment 0.8–1.0× length of second; female seventh sternite 2.0–2.8× length of sixth; male valva with three to four pectens along its ventral margin; female ovipositor apically acute or truncate. These modifications apply equally to the family Tridentaformidae, which was established for this genus in Regier et al. (2015).

### ﻿ Tridentaforma browncopper

Taxon classificationAnimaliaLepidopteraTridentaformidae

Monckton & Levesque-Beaudin sp. nov.

24742B6C-B00D-5A9C-91A5-2FC1BC0BCE08

https://zoobank.org/DC1D05C0-1D70-42F5-AD90-7E5EB0E8A702

**Figure 2.**
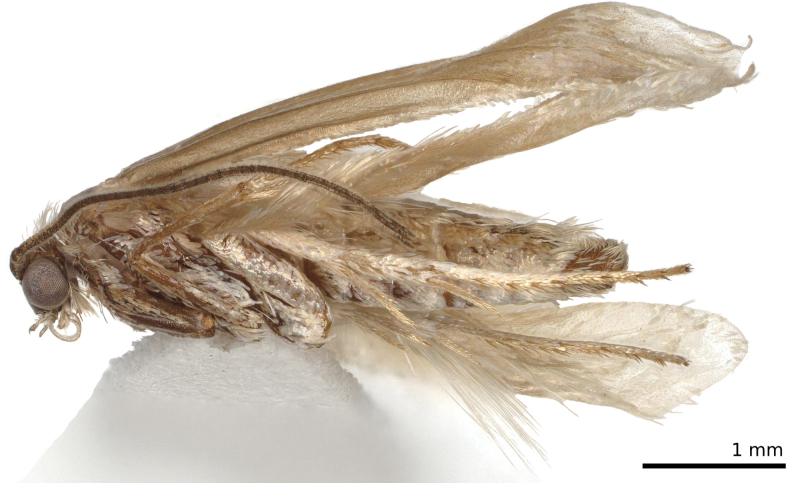
*Tridentaforma browncopper* Monckton & Levesque-Beaudin, sp. nov., male habitus, left lateral view (CBG-A17480-H03). Specimen was dried following storage in ethanol.

**Figure 3.**
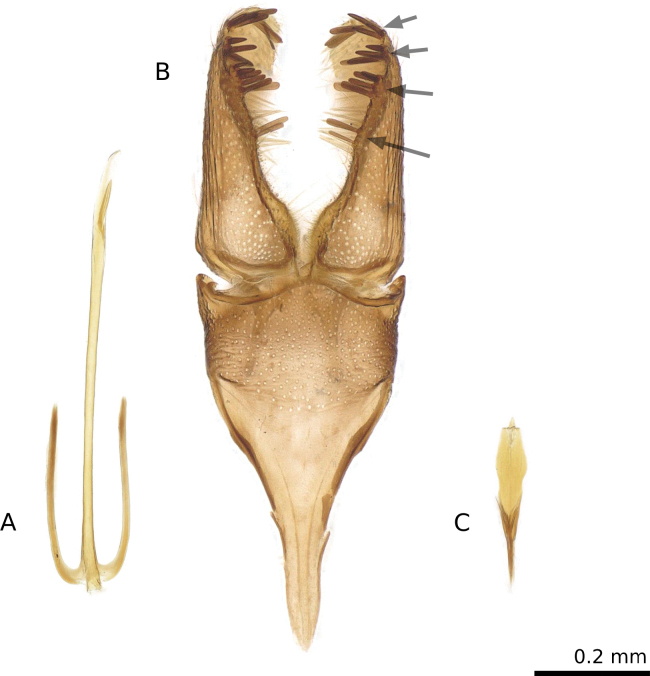
*Tridentaforma browncopper* Monckton & Levesque-Beaudin, sp. nov., male genitalia, ventral view. A. Phallus (CBG-A18330-D10); B. Genital capsule (CNCLEP00324919 / CBG-A18330-D04); C. Juxta (CBG-A18330-D10). The presence of four pectens (arrows) along the ventral margin of the valva distinguishes this species from *T. fuscoleuca* (Braun), which has only three.

**Figure 4.**
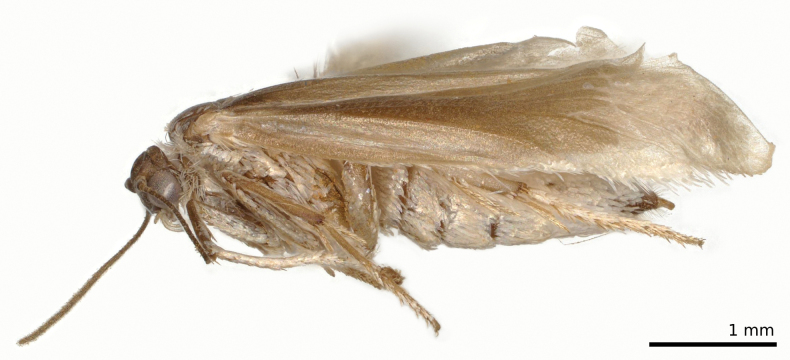
*Tridentaforma browncopper* Monckton & Levesque-Beaudin, sp. nov., female habitus, left lateral view (CBG-A17481-D01). Specimen was dried following storage in ethanol.

**Figure 5.**
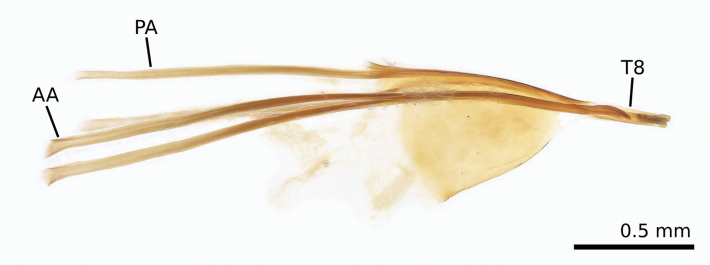
*Tridentaforma browncopper* Monckton & Levesque-Beaudin, sp. nov., female genitalia, left lateral view (CNCLEP00324920 / CBG-A18330-H08). The slightly widened, truncate apex of T8 and the anterior (AA) and posterior apophyses (PA) of similar length distinguish this species from *T. fuscoleuca* (Braun), in which T8 is narrower and subacute apically, and the anterior apophysis is distinctly shorter than the posterior.

**Figure 6.**
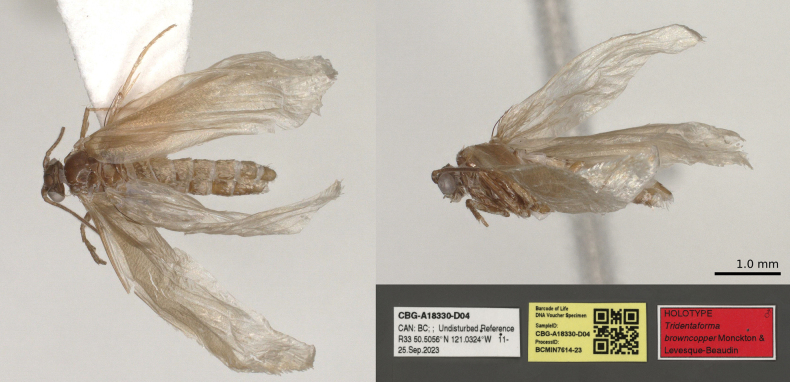
*Tridentaforma browncopper* Monckton & Levesque-Beaudin, sp. nov., holotype specimen and labels (CNCLEP00324919 / CBG-A18330-D04). Specimen was dried following storage in ethanol.

Fig. 2(male habitus); Fig. 3(male genitalia); Fig. 4(female habitus); Fig. 5(female genitalia); Fig. 6 (holotype specimen)

#### Material examined.

***Holotype***: Canada • ♂; British Columbia, Undisturbed Reference R33; 50.506°, −121.032°; 11–25.ix.2023; Malaise trap; CNCLEP00324919; CBG-A18330-D04. ***Allotype***: Canada • ♀; same data as holotype; CNCLEP00324920; CBG-A18330-H08. ***Paratypes***: Canada • 2♂; same data as holotype; CBG-A18330-D06, CBG-A18330-D10 • 1♂ 1♀; same locality as holotype; 29 Jul.–11 Sep. 2023; CBG-A17481-D01, CBG-A17481-D08 • 2♂ 2♀; British Columbia, Undisturbed Reference R20; 50.505°, −121.015°; 29 Aug.–11 Sep. 2023; CBG-A17480-E06, CBG-A17480-F11, CBG-A17480-H03, CBG-A17480-H11 • 4♂; same locality as preceding; 11–25 Sep. 2023; CBG-A18321-C01, CBG-A18321-E07, CBG-A18321-E08, CBG-A18321-F09 • 2♂ 1♀; same locality as preceding; 16–29 Aug. 2023; CBG-A16640-F01, CBG-A16640-G05, CBG-A16640-G12 • 4♂ 1♀; Undisturbed Reference R54, 50.53°, −121.059°; 28 Aug.–11 Sep. 2023; CBG-A17479-B07, CBG-A17479-B10, CBG-A17479-D02, CBG-A17479-D12, CBG-A17479-F07 • 2♀; same locality as preceding; 11–25 Sep. 2023; CBG-A18334-C11, CBG-A18334-F11. ***Other material***: see Suppl. material 1.

#### Diagnosis.

Females can be distinguished by the structure of their ovipositor (Fig. 5) with T8 slightly widened and broadly truncate apically (in *T. fuscoleuca* it is narrower and subacute apically), anterior and posterior apophyses approximately equal in length (in *T. fuscoleuca* the anterior apophysis is distinctly shorter than the posterior apophysis), although the latter character requires dissection to assess. Males are easily distinguished by the presence of four pectens along the ventral margin of the valva (Fig. 3), while *T. fuscoleuca* has three. The species can be identified by COI DNA barcoding as it possesses diagnostic substitutions at three nucleotide positions (118-C; 250-C; 277-A), which differentiate it from the other seven *Tridentaforma* BINs.

#### Description.

Small, slender-bodied moths; primarily brown with silvery, pale-brown scales; wing expanse 8.5–10.5 mm. ***Head***: antenna 40–44 segmented, simple, approximately 0.7–0.8× length of forewing. Compound eye moderately large, eye index approximately 1.1–1.2. Labial palp three-segmented with apical segment shorter (approximately 0.8× length of second). ***Thorax***: foretibia with pectinate epiphysis from middle, extending approximately halfway to apex. Forewing somewhat narrow, greatest width about 3.3× length. ***Abdomen***: female seventh sternite 2.0–2.4× length of sixth. ***Male genitalia***: uncus reduced, consisting of two small lobes. Vinculum and saccus well developed, saccus elongate and approximately Y-shaped, gradually tapering basally to about ¼ of its maximum apical width; total length 1.3× length of valva (Fig. 3B). Valva somewhat helical, relatively narrow in dorsal and ventral view, broad in lateral view; a series of four pectens spaced along its ventral margin, each of the apical three consisting of a short transverse row of 4–7 stout, spatulate spines, longer medially, the last 2–3 spines noticeably less-sclerotized and anteriorly directed; the ventral pecten consisting of 2 relatively long, less-sclerotized spines. Juxta (Fig. 3C) reduced in size, about 1/3 as long as median branch of phallus, and slender, produced anteriorly to a sharp point. Phallus (Fig. 3A) three-branched, median branch more than twice as long as lateral branches. ***Female genitalia***: apex of ovipositor slender, slightly flattened dorsoventrally, truncate, with smooth margins (Fig. 5). Anterior and posterior apophyses extremely slender and elongate, approximately equal in length to one another and about twice as long as T8. Proximal margin of T8 dorsally produced to an acute angle; apex of T8 broadly truncate and slightly widened relative to subapical constriction.

#### Etymology.

nłeʔképmx Elders, Knowledge Keepers, and people of the Citxw Nlaka’pamux Assembly chose skʷúnkʷl̓itkax̣n̓I as the name for this species, which means “brown copper moth”. Translation was required because the International Code of Zoological Nomenclature (International Commission on Zoological Nomenclature 1999) prohibits non-Latin characters. As a result, the CNA selected the species epithet “browncopper”, a compound noun in apposition formed from its English name.

#### Distribution.

This species is only known from the Thompson Plateau in south-central British Columbia, Canada.

#### Genetic data.

**Figure 7.**
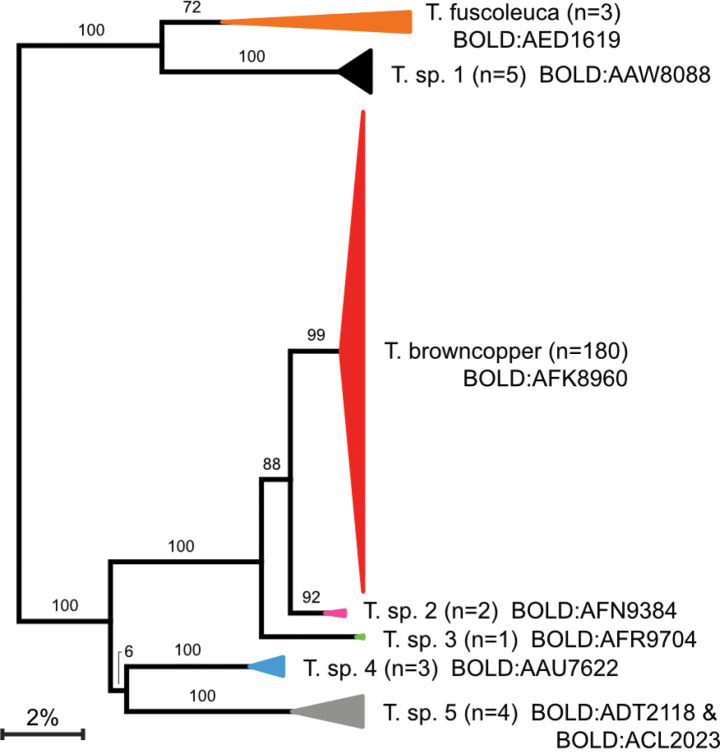
Simplified neighbor-joining tree from analysis of the 658 bp barcode region of COI-5P showing relationships between available DNA barcode records for Tridentaformidae. Coloured triangles indicate clusters corresponding to known or putative species of *Tridentaforma*; for each cluster, horizontal length indicates intraspecific divergence, and height reflects specimen count for each taxon (shown in parentheses). BINs associated with each cluster are indicated next to the putative species names. Numbers above branches are bootstrap support values (*n* = 100). The full tree is available in Suppl. material 2.

All members of this species belong to a single BIN (BOLD:AFK8960) with a maximum within species *p*-distance of 1.14%. The most closely related BIN (BOLD:AFN9384), an undescribed species from California (Fig. 7), is 2.72% divergent. DNA barcode records for specimens of *Tridentaforma* on BOLD are assigned to eight BINs (BOLD:AAW8088, BOLD:AED1619, BOLD:AFK8960, BOLD:AFN9384, BOLD:AFR9704, BOLD:ACL2023, BOLD:ADT2118, BOLD:AAU7622). Based on dissections and genetic data, we believe that five more species await description (Fig. 7). van Nieukerken and Davis (2023) noted five barcode clusters within Tridentaformidae which presumably correspond to the five BINs that were publicly accessible on BOLD at that time.

#### Biology.

Moths in the superfamily Adeloidea have various life histories, including leaf miners, skeletonizers, ground feeders, gallers, and borers in seeds, fruits, or stems (Regier et al. 2015). In California, adults of *Tridentaforma* fly around manzanita (*Arctostaphylos* spp., Ericaceae) in the early spring (van Nieukerken and Davis 2023) and have been observed ovipositing on the leaves of *Arctostaphylos tomentosa* Pursh (Detka et al. 2019). Detka et al. (2019) hypothesized that *Tridentaforma* larvae feed inside blister mines on *A. tomentosa*, but the organism that causes those mines has apparently not been determined. The species they observed appears to be crepuscular and active primarily at sundown, with at least two generations per year (Detka et al. 2019). Manzanita does not co-occur with *T. browncopper*; instead, the caterpillars may be concealed feeders on another ericaceous plant prevalent at the HVC reference site. All presently known adults of *T. browncopper* were collected in late August and September, which may represent a second or third generation of adults for the season.

#### Comments.

Davis (1978) noted “considerable variation” among specimens of *Tridentaforma* and suggested that a second species might occur among western populations of *T. fuscoleuca*. It is unclear if any of his specimens were from British Columbia, but two BINs (BOLD:AFN9384, BOLD:AAW8088) from California have several genitalia characters in common with *T. browncopper*.

## ﻿Discussion

This study resulted from a collaboration involving biodiversity scientists, industry representatives, and Indigenous communities. Support from Teck was critical to enable a biotic survey which delivered detailed baseline information on arthropod communities at HVC. Aside from providing Teck with the information required to better manage its restoration operations, the survey made a rapid and impactful contribution to science by advancing knowledge of species distributions and abundance. Although biotic surveys are often undertaken by mining firms to meet regulatory compliance, the resulting data often remains grey literature in the form of internal technical reports (Marques et al. 2024). As such, their impact on the broader scientific community is negligible. This study shows how Teck’s goal of advancing its understanding of biodiversity patterns at HVC was achieved while also making a net contribution to biodiversity science, with active participation by Indigenous communities. For example, the CNA viewed the opportunity to name the new species as, “a powerful act of cultural preservation and revitalization that acknowledges the importance of Indigenous Knowledge in biodiversity conservation” (CNA 2024). Indeed, following consultation with nłeʔképmx Elders and Knowledge Keepers, CNA highlighted a nłeʔkepmxcín phrase that references the relatively warm climate near HVC (“qʷəcqʷecúym̓x tk nkikax̣n̓í,” shared by Amelia Washington). This insight hints at a possible biogeographic scenario for the presence of *T. browncopper*, because apart from a putative species on Vancouver Island, the family is only known from southerly sites. The ancestors of skʷúnkʷl̓itkax̣n̓I could have persisted in the Thompson Region as surrounding areas cooled, leading to their separation from their southern counterparts.

Little is known about the life history of species in the genus *Tridentaforma*. In California, adults are associated with manzanita (*Arctostaphylos* spp., Ericaceae) and have been observed ovipositing on *Arctostaphylos tomentosa* Pursh, though these records may refer to an undescribed coastal species, as *T. fuscoleuca* was described from the Sierra Nevada, where coastal manzanitas are absent (Detka et al. 2019; van Nieukerken and Davis 2023; Calflora 2025). Detka and colleagues linked the coastal moths to blister mines on *A. tomentosa* leaves but have not apparently investigated further. Given the concealed feeding habits typical of Adeloidea (Regier et al. 2015) and the absence of manzanitas in south-central British Columbia, we suspect that *T. browncopper* larvae are leaf miners, fruit or stem borers, or otherwise-concealed feeders on another plant in the heather family (Ericaceae). Two species of *Vaccinium*—*V. membranaceum* Douglas ex Torr and *V. cespitosum* Michx.—occur at HVC and are plausible candidates, given their relatively restricted distributions. To date, adults have only been collected in late summer, but we expect *T. browncopper* to be at least bivoltine; our sampling in 2023–2024 began in July, likely missing earlier generations. Additional sampling began in May 2025, which may yield adults from earlier in the season. We also aim to locate larvae or signs of herbivory during upcoming fieldwork.

DNA barcoding (Hebert et al. 2003, 2004) is now widely used to accelerate species descriptions (Mutanen et al. 2013; Huemer et al. 2020; Lopez-Vaamonde et al. 2021). However, many species first revealed by DNA barcoding experience a long delay before their formal description. Although DNA barcodes are as effective if not better than morphology in defining species boundaries (Ortiz et al. 2017), descriptions based solely on DNA barcodes are often challenged (Ahrens et al. 2021; Engel et al. 2021). Yet, as rates of species extinction may already exceed those of species description (Costello et al. 2024), there is a clear need to adopt new protocols for species description. One approach (Kekkonen and Hebert 2014), which couples evidence of barcode divergence with abbreviated morphological characterization, was employed in this study of *Tridentaforma*. In our case, neither author involved in the species description had specialized knowledge of lepidopteran morphology or taxonomy; their expertise lies in other holometabolous insect taxa (SKM: Hymenoptera; VL-B: Diptera), but they are nevertheless suitably qualified to assess the available evidence. This approach is particularly effective when taxon diversity is low. An alternate approach will be essential in hyper-diverse groups such as Cecidomyiidae (Diptera) where it is likely that more than a million species await description (Hebert et al. 2016). In these cases, heavy reliance on DNA-based species delimitation is the only feasible approach, and evidence can be added by coupling COI barcoding with the targeted analysis of a few nuclear genes.

## Supplementary Material

XML Treatment for Tridentaforma

XML Treatment for Tridentaforma browncopper

## ﻿Additional information

### Use of AI

No use of AI was reported.

### Author ORCIDs

Spencer K. Monckton https://orcid.org/0000-0002-9879-9118

Valerie Levesque-Beaudin https://orcid.org/0000-0002-6053-0949

Ken A. Thompson https://orcid.org/0000-0002-1487-3254

Paul D. N. Hebert https://orcid.org/0000-0002-3081-6700

### Data availability

All of the data that support the findings of this study are available in the main text or Supplementary Information.

## Supplementary materials

Supplementary material 1 Tridentaformidae records from BOLD Data type csv Explanation note Tridentaformidae records from BOLD, provided in Barcode Core Data Model (BCDM) format. https://binary.pensoft.net/file/1448170This dataset is made available under the Open Database License (http://opendatacommons.org/licenses/odbl/1.0/). The Open Database License (ODbL) is a license agreement intended to allow users to freely share, modify, and use this Dataset while maintaining this same freedom for others, provided that the original source and author(s) are credited.Spencer K. Monckton, Valerie Levesque-Beaudin, Ken A. Thompson, Aidan Barnes, Kami French, Hunter Hampton, Paul D. N. Hebert

Supplementary material 2 Neighbour-joining tree Data type pdf Explanation note Neighbour-joining tree from analysis of COI-5P barcode records from 188 specimens of Tridentaformidae (BOLD dataset: DS-BRWNCP). https://binary.pensoft.net/file/1448171This dataset is made available under the Open Database License (http://opendatacommons.org/licenses/odbl/1.0/). The Open Database License (ODbL) is a license agreement intended to allow users to freely share, modify, and use this Dataset while maintaining this same freedom for others, provided that the original source and author(s) are credited.Spencer K. Monckton, Valerie Levesque-Beaudin, Ken A. Thompson, Aidan Barnes, Kami French, Hunter Hampton, Paul D. N. Hebert
