# Supplementary material for: ﻿The brown copper moth, Tridentaforma browncopper: DNA barcoding reveals a second species in the family Tridentaformidae (Lepidoptera, Adeloidea)
Source: Zookeys. 2025 Oct 24;1257:25–38. doi: 10.3897/zookeys.1257.158827 (PMC12579331; doi:10.3897/zookeys.1257.158827)
Supplement: Supplementary material 2 — Neighbour-joining tree [file zookeys-1257-025_article-158827__-s002.pdf]

# BOLD TaxonID Tree

| | | |
|---|---|---|
| Title | : | Tree Result - DS-BRWNCP (199 records selected) |
| Date | : | 13-Aug-2025 |
| Data Type | : | Nucleotide |
| Distance Model | : | Kimura 2 Parameter |
| Marker | : | COI-5P |
| Colourization | : | [blue]=Stop Codons [red]=Contamination or misidentification |

| | | |
|---|---|---|
| Label | : | Sample ID |
| Label | : | Process ID |
| Label | : | Taxon |
| Label | : | Province/State |
| Label | : | Barcode Cluster (BIN) |

| | | |
|---|---|---|
| Sequence Count | : | 198 |
| Species count | : | 1 |
| Genus count | : | 1 |
| Family count | : | 1 |
| Unidentified | : | 193 |

| | | |
|---|---|---|
| BIN Count | : | 8 |

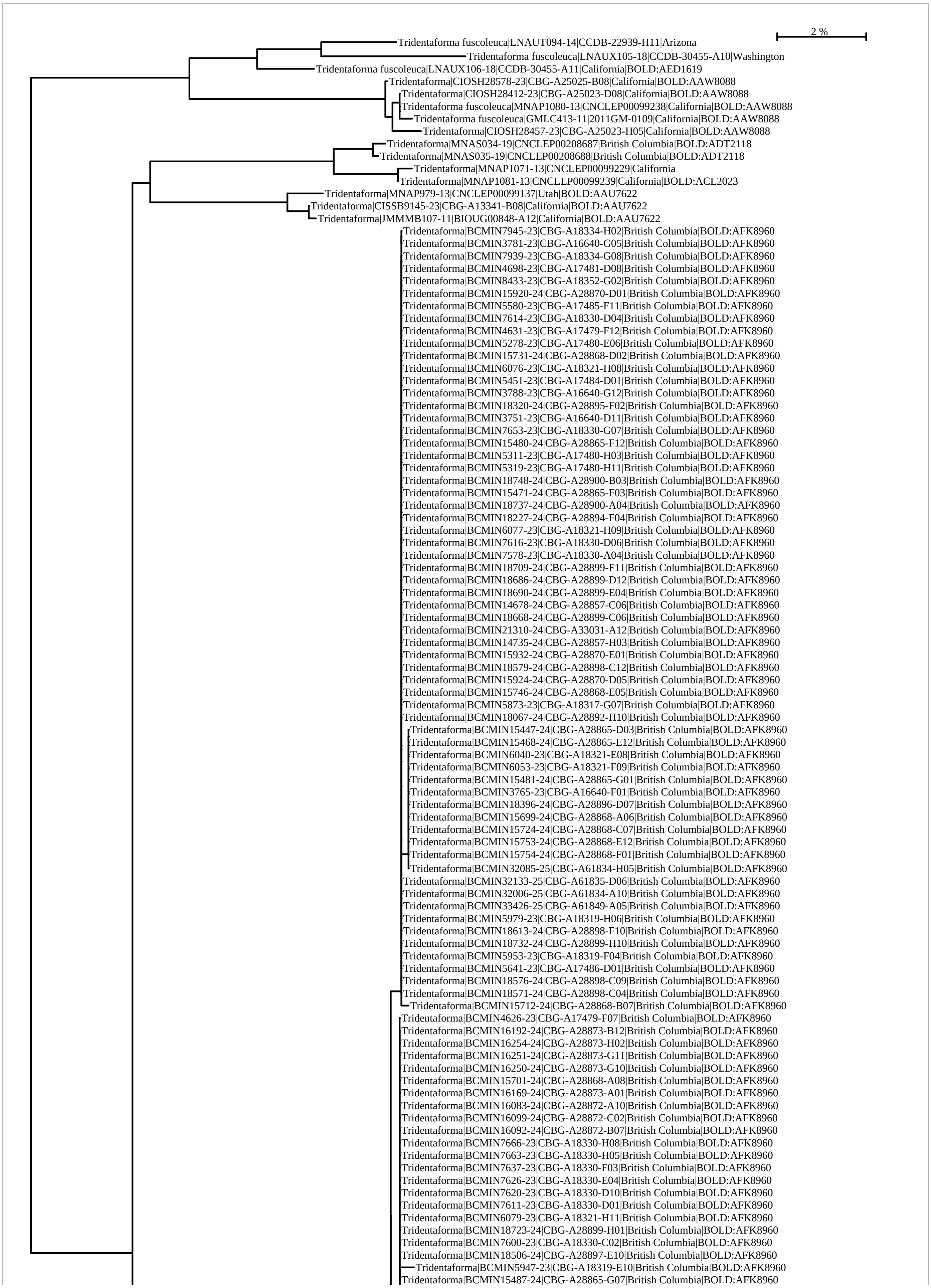
2 %
Tridentaforma fuscoleuca|LNAUT094-14|CCDB-22939-H11|Arizona
Tridentaforma fuscoleuca|LNAUX105-18|CCDB-30455-A10|Washington
Tridentaforma fuscoleuca|LNAUX106-18|CCDB-30455-A11|California|BOLD:AED1619
Tridentaforma|CIOSH28578-23|CBG-A25025-B08|California|BOLD:AAW8088
Tridentaforma|CIOSH28412-23|CBG-A25023-D08|California|BOLD:AAW8088
Tridentaforma fuscoleuca|MNAP1080-13|CNCLEP00099238|California|BOLD:AAW8088
Tridentaforma fuscoleuca|GMLC413-11|2011GM-0109|California|BOLD:AAW8088
Tridentaforma|CIOSH28457-23|CBG-A25023-H05|California|BOLD:AAW8088
Tridentaforma|MNAS034-19|CNCLEP00208687|British Columbia|BOLD:ADT2118
Tridentaforma|MNAS035-19|CNCLEP00208688|British Columbia|BOLD:ADT2118
Tridentaforma|MNAP1071-13|CNCLEP00099229|California
Tridentaforma|MNAP1081-13|CNCLEP00099239|California|BOLD:ACL2023
Tridentaforma|MNAP979-13|CNCLEP00099137|Utah|BOLD:AAU7622
Tridentaforma|CISSB9145-23|CBG-A13341-B08|California|BOLD:AAU7622
Tridentaforma|JMMMB107-11|BIOUG00848-A12|California|BOLD:AAU7622
Tridentaforma|BCMIN7945-23|CBG-A18334-H02|British Columbia|BOLD:AFK8960
Tridentaforma|BCMIN3781-23|CBG-A16640-G05|British Columbia|BOLD:AFK8960
Tridentaforma|BCMIN7939-23|CBG-A18334-G08|British Columbia|BOLD:AFK8960
Tridentaforma|BCMIN4698-23|CBG-A17481-D08|British Columbia|BOLD:AFK8960
Tridentaforma|BCMIN8433-23|CBG-A18352-G02|British Columbia|BOLD:AFK8960
Tridentaforma|BCMIN15920-24|CBG-A28870-D01|British Columbia|BOLD:AFK8960
Tridentaforma|BCMIN5580-23|CBG-A17485-F11|British Columbia|BOLD:AFK8960
Tridentaforma|BCMIN7614-23|CBG-A18330-D04|British Columbia|BOLD:AFK8960
Tridentaforma|BCMIN4631-23|CBG-A17479-F12|British Columbia|BOLD:AFK8960
Tridentaforma|BCMIN5278-23|CBG-A17480-E06|British Columbia|BOLD:AFK8960
Tridentaforma|BCMIN15731-24|CBG-A28868-D02|British Columbia|BOLD:AFK8960
Tridentaforma|BCMIN6076-23|CBG-A18321-H08|British Columbia|BOLD:AFK8960
Tridentaforma|BCMIN5451-23|CBG-A17484-D01|British Columbia|BOLD:AFK8960
Tridentaforma|BCMIN3788-23|CBG-A16640-G12|British Columbia|BOLD:AFK8960
Tridentaforma|BCMIN18320-24|CBG-A28895-F02|British Columbia|BOLD:AFK8960
Tridentaforma|BCMIN3751-23|CBG-A16640-D11|British Columbia|BOLD:AFK8960
Tridentaforma|BCMIN7653-23|CBG-A18330-G07|British Columbia|BOLD:AFK8960
Tridentaforma|BCMIN15480-24|CBG-A28865-F12|British Columbia|BOLD:AFK8960
Tridentaforma|BCMIN5311-23|CBG-A17480-H03|British Columbia|BOLD:AFK8960
Tridentaforma|BCMIN5319-23|CBG-A17480-H11|British Columbia|BOLD:AFK8960
Tridentaforma|BCMIN18748-24|CBG-A28900-B03|British Columbia|BOLD:AFK8960
Tridentaforma|BCMIN15471-24|CBG-A28865-F03|British Columbia|BOLD:AFK8960
Tridentaforma|BCMIN18737-24|CBG-A28900-A04|British Columbia|BOLD:AFK8960
Tridentaforma|BCMIN18227-24|CBG-A28894-F04|British Columbia|BOLD:AFK8960
Tridentaforma|BCMIN6077-23|CBG-A18321-H09|British Columbia|BOLD:AFK8960
Tridentaforma|BCMIN7616-23|CBG-A18330-D06|British Columbia|BOLD:AFK8960
Tridentaforma|BCMIN7578-23|CBG-A18330-A04|British Columbia|BOLD:AFK8960
Tridentaforma|BCMIN18709-24|CBG-A28899-F11|British Columbia|BOLD:AFK8960
Tridentaforma|BCMIN18686-24|CBG-A28899-D12|British Columbia|BOLD:AFK8960
Tridentaforma|BCMIN18690-24|CBG-A28899-E04|British Columbia|BOLD:AFK8960
Tridentaforma|BCMIN14678-24|CBG-A28857-C06|British Columbia|BOLD:AFK8960
Tridentaforma|BCMIN18668-24|CBG-A28899-C06|British Columbia|BOLD:AFK8960
Tridentaforma|BCMIN21310-24|CBG-A33031-A12|British Columbia|BOLD:AFK8960
Tridentaforma|BCMIN14735-24|CBG-A28857-H03|British Columbia|BOLD:AFK8960
Tridentaforma|BCMIN15932-24|CBG-A28870-E01|British Columbia|BOLD:AFK8960
Tridentaforma|BCMIN18579-24|CBG-A28898-C12|British Columbia|BOLD:AFK8960
Tridentaforma|BCMIN15924-24|CBG-A28870-D05|British Columbia|BOLD:AFK8960
Tridentaforma|BCMIN15746-24|CBG-A28868-E05|British Columbia|BOLD:AFK8960
Tridentaforma|BCMIN5873-23|CBG-A18317-G07|British Columbia|BOLD:AFK8960
Tridentaforma|BCMIN18067-24|CBG-A28892-H10|British Columbia|BOLD:AFK8960
Tridentaforma|BCMIN15447-24|CBG-A28865-D03|British Columbia|BOLD:AFK8960
Tridentaforma|BCMIN15468-24|CBG-A28865-E12|British Columbia|BOLD:AFK8960
Tridentaforma|BCMIN6040-23|CBG-A18321-E08|British Columbia|BOLD:AFK8960
Tridentaforma|BCMIN6053-23|CBG-A18321-F09|British Columbia|BOLD:AFK8960
Tridentaforma|BCMIN15481-24|CBG-A28865-G01|British Columbia|BOLD:AFK8960
Tridentaforma|BCMIN3765-23|CBG-A16640-F01|British Columbia|BOLD:AFK8960
Tridentaforma|BCMIN18396-24|CBG-A28896-D07|British Columbia|BOLD:AFK8960
Tridentaforma|BCMIN15699-24|CBG-A28868-A06|British Columbia|BOLD:AFK8960
Tridentaforma|BCMIN15724-24|CBG-A28868-C07|British Columbia|BOLD:AFK8960
Tridentaforma|BCMIN15753-24|CBG-A28868-E12|British Columbia|BOLD:AFK8960
Tridentaforma|BCMIN15754-24|CBG-A28868-F01|British Columbia|BOLD:AFK8960
Tridentaforma|BCMIN32085-25|CBG-A61834-H05|British Columbia|BOLD:AFK8960
Tridentaforma|BCMIN32133-25|CBG-A61835-D06|British Columbia|BOLD:AFK8960
Tridentaforma|BCMIN32006-25|CBG-A61834-A10|British Columbia|BOLD:AFK8960
Tridentaforma|BCMIN33426-25|CBG-A61849-A05|British Columbia|BOLD:AFK8960
Tridentaforma|BCMIN5979-23|CBG-A18319-H06|British Columbia|BOLD:AFK8960
Tridentaforma|BCMIN18613-24|CBG-A28898-F10|British Columbia|BOLD:AFK8960
Tridentaforma|BCMIN18732-24|CBG-A28899-H10|British Columbia|BOLD:AFK8960
Tridentaforma|BCMIN5953-23|CBG-A18319-F04|British Columbia|BOLD:AFK8960
Tridentaforma|BCMIN5641-23|CBG-A17486-D01|British Columbia|BOLD:AFK8960
Tridentaforma|BCMIN18576-24|CBG-A28898-C09|British Columbia|BOLD:AFK8960
Tridentaforma|BCMIN18571-24|CBG-A28898-C04|British Columbia|BOLD:AFK8960
Tridentaforma|BCMIN15712-24|CBG-A28868-B07|British Columbia|BOLD:AFK8960
Tridentaforma|BCMIN4626-23|CBG-A17479-F07|British Columbia|BOLD:AFK8960
Tridentaforma|BCMIN16192-24|CBG-A28873-B12|British Columbia|BOLD:AFK8960
Tridentaforma|BCMIN16254-24|CBG-A28873-H02|British Columbia|BOLD:AFK8960
Tridentaforma|BCMIN16251-24|CBG-A28873-G11|British Columbia|BOLD:AFK8960
Tridentaforma|BCMIN16250-24|CBG-A28873-G10|British Columbia|BOLD:AFK8960
Tridentaforma|BCMIN15701-24|CBG-A28868-A08|British Columbia|BOLD:AFK8960
Tridentaforma|BCMIN16169-24|CBG-A28873-A01|British Columbia|BOLD:AFK8960
Tridentaforma|BCMIN16083-24|CBG-A28872-A10|British Columbia|BOLD:AFK8960
Tridentaforma|BCMIN16099-24|CBG-A28872-C02|British Columbia|BOLD:AFK8960
Tridentaforma|BCMIN16092-24|CBG-A28872-B07|British Columbia|BOLD:AFK8960
Tridentaforma|BCMIN7666-23|CBG-A18330-H08|British Columbia|BOLD:AFK8960
Tridentaforma|BCMIN7663-23|CBG-A18330-H05|British Columbia|BOLD:AFK8960
Tridentaforma|BCMIN7637-23|CBG-A18330-F03|British Columbia|BOLD:AFK8960
Tridentaforma|BCMIN7626-23|CBG-A18330-E04|British Columbia|BOLD:AFK8960
Tridentaforma|BCMIN7620-23|CBG-A18330-D10|British Columbia|BOLD:AFK8960
Tridentaforma|BCMIN7611-23|CBG-A18330-D01|British Columbia|BOLD:AFK8960
Tridentaforma|BCMIN6079-23|CBG-A18321-H11|British Columbia|BOLD:AFK8960
Tridentaforma|BCMIN18723-24|CBG-A28899-H01|British Columbia|BOLD:AFK8960
Tridentaforma|BCMIN7600-23|CBG-A18330-C02|British Columbia|BOLD:AFK8960
Tridentaforma|BCMIN18506-24|CBG-A28897-E10|British Columbia|BOLD:AFK8960
Tridentaforma|BCMIN5947-23|CBG-A18319-E10|British Columbia|BOLD:AFK8960
Tridentaforma|BCMIN15487-24|CBG-A28865-G07|British Columbia|BOLD:AFK8960

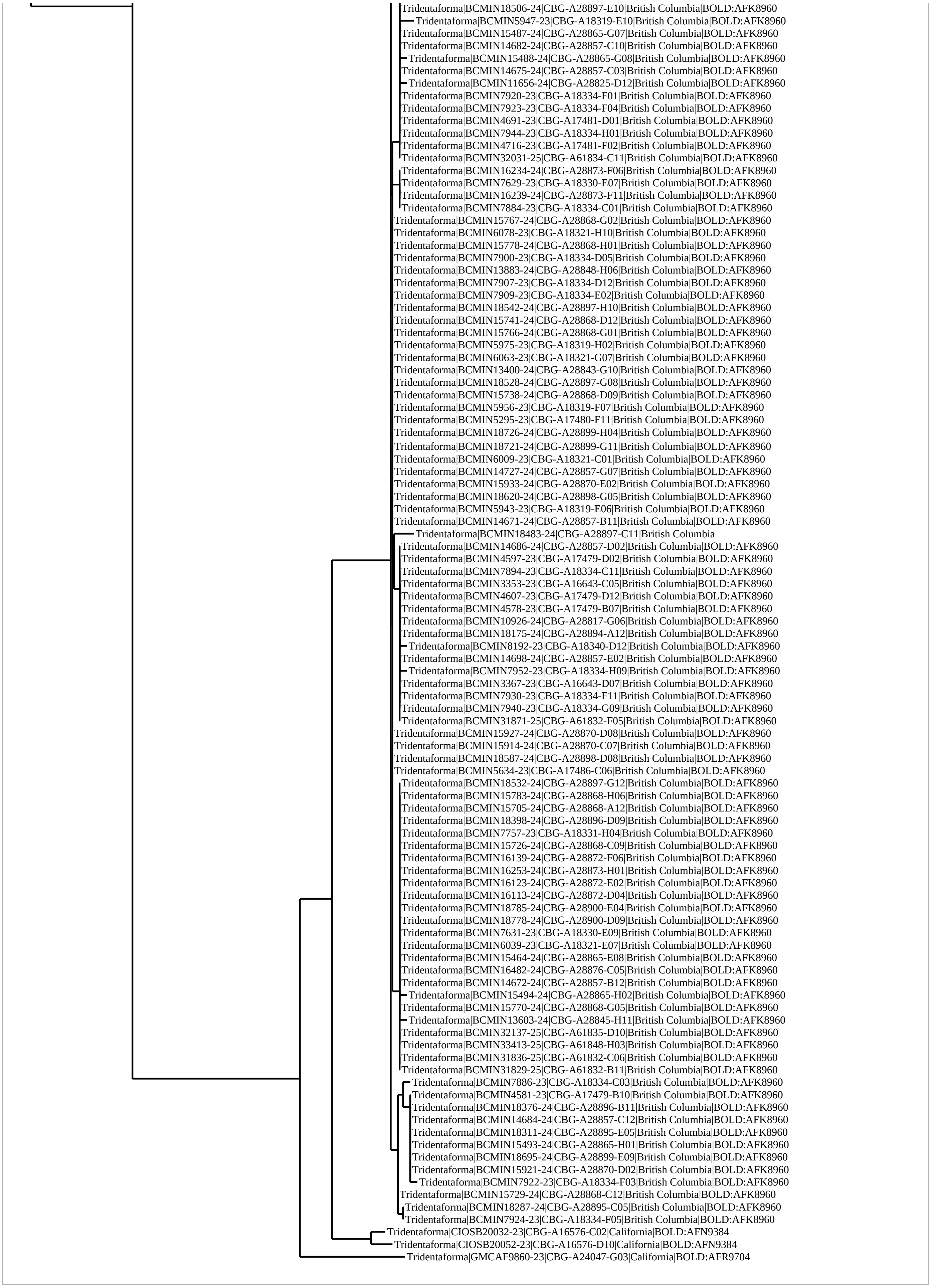
Tridentaforma|BCMIN18506-24|CBG-A28897-E10|British Columbia|BOLD:AFK8960
Tridentaforma|BCMIN5947-23|CBG-A18319-E10|British Columbia|BOLD:AFK8960
Tridentaforma|BCMIN15487-24|CBG-A28865-G07|British Columbia|BOLD:AFK8960
Tridentaforma|BCMIN14682-24|CBG-A28857-C10|British Columbia|BOLD:AFK8960
Tridentaforma|BCMIN15488-24|CBG-A28865-G08|British Columbia|BOLD:AFK8960
Tridentaforma|BCMIN14675-24|CBG-A28857-C03|British Columbia|BOLD:AFK8960
Tridentaforma|BCMIN11656-24|CBG-A28825-D12|British Columbia|BOLD:AFK8960
Tridentaforma|BCMIN7920-23|CBG-A18334-F01|British Columbia|BOLD:AFK8960
Tridentaforma|BCMIN7923-23|CBG-A18334-F04|British Columbia|BOLD:AFK8960
Tridentaforma|BCMIN4691-23|CBG-A17481-D01|British Columbia|BOLD:AFK8960
Tridentaforma|BCMIN7944-23|CBG-A18334-H01|British Columbia|BOLD:AFK8960
Tridentaforma|BCMIN4716-23|CBG-A17481-F02|British Columbia|BOLD:AFK8960
Tridentaforma|BCMIN32031-25|CBG-A61834-C11|British Columbia|BOLD:AFK8960
Tridentaforma|BCMIN16234-24|CBG-A28873-F06|British Columbia|BOLD:AFK8960
Tridentaforma|BCMIN7629-23|CBG-A18330-E07|British Columbia|BOLD:AFK8960
Tridentaforma|BCMIN16239-24|CBG-A28873-F11|British Columbia|BOLD:AFK8960
Tridentaforma|BCMIN7884-23|CBG-A18334-C01|British Columbia|BOLD:AFK8960
Tridentaforma|BCMIN15767-24|CBG-A28868-G02|British Columbia|BOLD:AFK8960
Tridentaforma|BCMIN6078-23|CBG-A18321-H10|British Columbia|BOLD:AFK8960
Tridentaforma|BCMIN15778-24|CBG-A28868-H01|British Columbia|BOLD:AFK8960
Tridentaforma|BCMIN7900-23|CBG-A18334-D05|British Columbia|BOLD:AFK8960
Tridentaforma|BCMIN13883-24|CBG-A28848-H06|British Columbia|BOLD:AFK8960
Tridentaforma|BCMIN7907-23|CBG-A18334-D12|British Columbia|BOLD:AFK8960
Tridentaforma|BCMIN7909-23|CBG-A18334-E02|British Columbia|BOLD:AFK8960
Tridentaforma|BCMIN18542-24|CBG-A28897-H10|British Columbia|BOLD:AFK8960
Tridentaforma|BCMIN15741-24|CBG-A28868-D12|British Columbia|BOLD:AFK8960
Tridentaforma|BCMIN15766-24|CBG-A28868-G01|British Columbia|BOLD:AFK8960
Tridentaforma|BCMIN5975-23|CBG-A18319-H02|British Columbia|BOLD:AFK8960
Tridentaforma|BCMIN6063-23|CBG-A18321-G07|British Columbia|BOLD:AFK8960
Tridentaforma|BCMIN13400-24|CBG-A28843-G10|British Columbia|BOLD:AFK8960
Tridentaforma|BCMIN18528-24|CBG-A28897-G08|British Columbia|BOLD:AFK8960
Tridentaforma|BCMIN15738-24|CBG-A28868-D09|British Columbia|BOLD:AFK8960
Tridentaforma|BCMIN5956-23|CBG-A18319-F07|British Columbia|BOLD:AFK8960
Tridentaforma|BCMIN5295-23|CBG-A17480-F11|British Columbia|BOLD:AFK8960
Tridentaforma|BCMIN18726-24|CBG-A28899-H04|British Columbia|BOLD:AFK8960
Tridentaforma|BCMIN18721-24|CBG-A28899-G11|British Columbia|BOLD:AFK8960
Tridentaforma|BCMIN6009-23|CBG-A18321-C01|British Columbia|BOLD:AFK8960
Tridentaforma|BCMIN14727-24|CBG-A28857-G07|British Columbia|BOLD:AFK8960
Tridentaforma|BCMIN15933-24|CBG-A28870-E02|British Columbia|BOLD:AFK8960
Tridentaforma|BCMIN18620-24|CBG-A28898-G05|British Columbia|BOLD:AFK8960
Tridentaforma|BCMIN5943-23|CBG-A18319-E06|British Columbia|BOLD:AFK8960
Tridentaforma|BCMIN14671-24|CBG-A28857-B11|British Columbia|BOLD:AFK8960
Tridentaforma|BCMIN18483-24|CBG-A28897-C11|British Columbia
Tridentaforma|BCMIN14686-24|CBG-A28857-D02|British Columbia|BOLD:AFK8960
Tridentaforma|BCMIN4597-23|CBG-A17479-D02|British Columbia|BOLD:AFK8960
Tridentaforma|BCMIN7894-23|CBG-A18334-C11|British Columbia|BOLD:AFK8960
Tridentaforma|BCMIN3353-23|CBG-A16643-C05|British Columbia|BOLD:AFK8960
Tridentaforma|BCMIN4607-23|CBG-A17479-D12|British Columbia|BOLD:AFK8960
Tridentaforma|BCMIN4578-23|CBG-A17479-B07|British Columbia|BOLD:AFK8960
Tridentaforma|BCMIN10926-24|CBG-A28817-G06|British Columbia|BOLD:AFK8960
Tridentaforma|BCMIN18175-24|CBG-A28894-A12|British Columbia|BOLD:AFK8960
Tridentaforma|BCMIN8192-23|CBG-A18340-D12|British Columbia|BOLD:AFK8960
Tridentaforma|BCMIN14698-24|CBG-A28857-E02|British Columbia|BOLD:AFK8960
Tridentaforma|BCMIN7952-23|CBG-A18334-H09|British Columbia|BOLD:AFK8960
Tridentaforma|BCMIN3367-23|CBG-A16643-D07|British Columbia|BOLD:AFK8960
Tridentaforma|BCMIN7930-23|CBG-A18334-F11|British Columbia|BOLD:AFK8960
Tridentaforma|BCMIN7940-23|CBG-A18334-G09|British Columbia|BOLD:AFK8960
Tridentaforma|BCMIN31871-25|CBG-A61832-F05|British Columbia|BOLD:AFK8960
Tridentaforma|BCMIN15927-24|CBG-A28870-D08|British Columbia|BOLD:AFK8960
Tridentaforma|BCMIN15914-24|CBG-A28870-C07|British Columbia|BOLD:AFK8960
Tridentaforma|BCMIN18587-24|CBG-A28898-D08|British Columbia|BOLD:AFK8960
Tridentaforma|BCMIN5634-23|CBG-A17486-C06|British Columbia|BOLD:AFK8960
Tridentaforma|BCMIN18532-24|CBG-A28897-G12|British Columbia|BOLD:AFK8960
Tridentaforma|BCMIN15783-24|CBG-A28868-H06|British Columbia|BOLD:AFK8960
Tridentaforma|BCMIN15705-24|CBG-A28868-A12|British Columbia|BOLD:AFK8960
Tridentaforma|BCMIN18398-24|CBG-A28896-D09|British Columbia|BOLD:AFK8960
Tridentaforma|BCMIN7757-23|CBG-A18331-H04|British Columbia|BOLD:AFK8960
Tridentaforma|BCMIN15726-24|CBG-A28868-C09|British Columbia|BOLD:AFK8960
Tridentaforma|BCMIN16139-24|CBG-A28872-F06|British Columbia|BOLD:AFK8960
Tridentaforma|BCMIN16253-24|CBG-A28873-H01|British Columbia|BOLD:AFK8960
Tridentaforma|BCMIN16123-24|CBG-A28872-E02|British Columbia|BOLD:AFK8960
Tridentaforma|BCMIN16113-24|CBG-A28872-D04|British Columbia|BOLD:AFK8960
Tridentaforma|BCMIN18785-24|CBG-A28900-E04|British Columbia|BOLD:AFK8960
Tridentaforma|BCMIN18778-24|CBG-A28900-D09|British Columbia|BOLD:AFK8960
Tridentaforma|BCMIN7631-23|CBG-A18330-E09|British Columbia|BOLD:AFK8960
Tridentaforma|BCMIN6039-23|CBG-A18321-E07|British Columbia|BOLD:AFK8960
Tridentaforma|BCMIN15464-24|CBG-A28865-E08|British Columbia|BOLD:AFK8960
Tridentaforma|BCMIN16482-24|CBG-A28876-C05|British Columbia|BOLD:AFK8960
Tridentaforma|BCMIN14672-24|CBG-A28857-B12|British Columbia|BOLD:AFK8960
Tridentaforma|BCMIN15494-24|CBG-A28865-H02|British Columbia|BOLD:AFK8960
Tridentaforma|BCMIN15770-24|CBG-A28868-G05|British Columbia|BOLD:AFK8960
Tridentaforma|BCMIN13603-24|CBG-A28845-H11|British Columbia|BOLD:AFK8960
Tridentaforma|BCMIN32137-25|CBG-A61835-D10|British Columbia|BOLD:AFK8960
Tridentaforma|BCMIN33413-25|CBG-A61848-H03|British Columbia|BOLD:AFK8960
Tridentaforma|BCMIN31836-25|CBG-A61832-C06|British Columbia|BOLD:AFK8960
Tridentaforma|BCMIN31829-25|CBG-A61832-B11|British Columbia|BOLD:AFK8960
Tridentaforma|BCMIN7886-23|CBG-A18334-C03|British Columbia|BOLD:AFK8960
Tridentaforma|BCMIN4581-23|CBG-A17479-B10|British Columbia|BOLD:AFK8960
Tridentaforma|BCMIN18376-24|CBG-A28896-B11|British Columbia|BOLD:AFK8960
Tridentaforma|BCMIN14684-24|CBG-A28857-C12|British Columbia|BOLD:AFK8960
Tridentaforma|BCMIN18311-24|CBG-A28895-E05|British Columbia|BOLD:AFK8960
Tridentaforma|BCMIN15493-24|CBG-A28865-H01|British Columbia|BOLD:AFK8960
Tridentaforma|BCMIN18695-24|CBG-A28899-E09|British Columbia|BOLD:AFK8960
Tridentaforma|BCMIN15921-24|CBG-A28870-D02|British Columbia|BOLD:AFK8960
Tridentaforma|BCMIN7922-23|CBG-A18334-F03|British Columbia|BOLD:AFK8960
Tridentaforma|BCMIN15729-24|CBG-A28868-C12|British Columbia|BOLD:AFK8960
Tridentaforma|BCMIN18287-24|CBG-A28895-C05|British Columbia|BOLD:AFK8960
Tridentaforma|BCMIN7924-23|CBG-A18334-F05|British Columbia|BOLD:AFK8960
Tridentaforma|CIOSB20032-23|CBG-A16576-C02|California|BOLD:AFN9384
Tridentaforma|CIOSB20052-23|CBG-A16576-D10|California|BOLD:AFN9384
Tridentaforma|GMCAF9860-23|CBG-A24047-G03|California|BOLD:AFR9704
